# Short-term pegylated interferon alpha in chronic HBV patients with ultra-low HBsAg: a retrospective study

**DOI:** 10.3389/fcimb.2025.1582997

**Published:** 2025-09-05

**Authors:** Huimin Liu, Hongmei Gong, Zhaoxia Tan, Yanyan Wu, Lijian Ran, Qing Mao, Guohong Deng, Li Jiang, Jie Xia

**Affiliations:** Department of Infectious Diseases, Southwest Hospital, Third Military Medical University (Army Medical University), Key Laboratory of Infectious Disease Research of Chongqing, Infectious Disease Clinical Research Center of Chongqing, Chongqing, China

**Keywords:** chronic hepatitis B, HBsAg clearance, ultra-low surface antigen levels, pegylated interferon, short-term therapy

## Abstract

**Background and aims:**

Hepatitis B surface antigen (HBsAg) clearance—defined as an HBsAg level below the lower limit of detection—is critical for the functional cure of chronic hepatitis B (CHB). This study evaluated the efficacy of short-term pegylated interferon alpha (Peg-IFNα) therapy in achieving HBsAg clearance in CHB patients with ultra-low HBsAg levels (<50 IU/ml).

**Methods:**

A total of 378 CHB patients with HBsAg levels <50 IU/ml were enrolled, including 206 nucleos(t)ide analogue (NUC)-treated patients and 172 inactive HBsAg carriers (IHCs). The NUC-treated cohort was divided into 83 patients receiving additional Peg-IFNα treatment (NUC add-on Peg-IFNα group) and 123 patients continuing NUC monotherapy (NUC group). The IHC cohort was divided into 86 patients receiving Peg-IFNα treatment (Peg-IFNα group) and 86 untreated patients (untreated group). The primary endpoint was the HBsAg clearance rate at week 24.

**Results:**

At week 24, the HBsAg clearance rates in the NUC add-on Peg-IFNα group and Peg-IFNα group were 69.88% and 55.81%, respectively (p = 0.059), both significantly higher than the zero clearance rates in the NUC and untreated groups (p < 0.001). Patients with baseline HBsAg <10 IU/ml achieved higher clearance rates [81.82% vs. 73.81% (p = 0.144)]. A decline of ≥95.8% in HBsAg levels from baseline to week 12 predicted HBsAg clearance at week 24 (AUC ≥0.9, sensitivity 0.765, specificity 0.961).

**Conclusions:**

Short-term Peg-IFNα therapy achieved high and comparable HBsAg clearance rates within 24 weeks in NUC-treated patients and IHCs with ultra-low HBsAg levels.

**Clinical trial registration:**

https://www.medicalresearch.org.cn/login, identifier MR-50-24-011565.

## Highlights

- Clearance of HBsAg is key to curing CHB.
- In CHB patients with low HBsAg levels (<50 IU/ml)—whether previously treated with NUC or IHCs (hepatitis B e antigen [HBeAg]-negative, with low or undetectable HBV DNA levels and normal alanine aminotransferase [ALT] levels)—Peg-IFNα therapy within 24 weeks achieved high HBsAg clearance rates.
- CHB patients with low levels of HBsAg should receive early intervention to achieve HBsAg clearance and improve clinical outcomes.

## 1 Introduction

Hepatitis B virus (HBV) infection is a major global public health issue. In 2022, approximately 254 million individuals worldwide were chronically infected with HBV, leading to about 1.1 million deaths, primarily from HBV-related cirrhosis and hepatocellular carcinoma (HCC) (World Health Organization, 2024; Hsu et al., 2023). Antiviral therapy is an effective strategy for preventing and managing complications in chronic hepatitis B (CHB) patients. Under the current limitations in completely eradicating HBV, achieving hepatitis B surface antigen (HBsAg) clearance (with or without seroconversion) is key to reducing the incidence of end-stage liver disease and mortality. This is considered a functional or clinical cure and is the ideal endpoint of antiviral therapy for CHB patients (Kaur et al., 2022; Vittal et al., 2022). However, enabling more CHB patients to achieve HBsAg clearance remains a formidable challenge.

The annual spontaneous HBsAg clearance rate is about 1%-2.4% (Lok et al., 2017; Cao et al., 2017; Zhou et al., 2019). For CHB patients undergoing nucleos(t)ide analogue (NUC) antiviral therapy, the HBsAg clearance rate is low, usually 0-3% (Hall et al., 2022; Hirode et al., 2022). Clinical findings show that in hepatitis e surface antigen (HBeAg)-negative CHB patients with HBsAg level ≤1,500 IU/ml following NUC treatment (the so-called advantaged population), the HBsAg clearance rate can reach 22.0%-33.2% after 48 weeks of sequential or combined pegylated interferon alpha (Peg-IFNα) therapy (Han et al., 2016; Ning et al., 2014; Hu et al., 2018). Evidence-based studies indicate that adding Peg-IFNα to NUC therapy for patients with low HBsAg levels can promote HBsAg clearance. However, there is no standardized protocol for patient selection or treatment duration. Furthermore, debate continues on whether to start antiviral treatment in inactive hepatitis B surface antigen carriers (IHCs), who are usually HBeAg-negative with low or undetectable HBV DNA levels, normal alanine aminotransferase (ALT) levels, and minimal or no liver inflammation (Sarin et al., 2016; European Association for the Study of the Liver, 2017; Terrault et al., 2018; Chinese Society of Infectious Diseases, Chinese Medical Association and Chinese Society of Hepatology, Chinese Medical Association, 2019).

Despite slow disease progression, IHCs retain a risk of histological disease progression (Zhuang, 2021). The annual hepatitis recurrence rate in IHCs is 1.1% (Bortolotti et al., 2006; Iorio et al., 2007). A study found that over an average 13.1-year follow- up, the risks of HCC and liver disease-related death in IHCs were 4.6-fold and 2.1-fold higher, respectively, compared with those in the general population (Chen et al., 2010). Moreover, the HCC risk in Asian IHCs is 10-fold higher than in European IHCs (Fattovich et al., 2008). A recent meta-analysis showed an overall HBsAg clearance rate of 47% in IHCs receiving 48-week Peg-IFNα treatment (Song et al., 2021a). Thus, a proactive approach to intervention might be prudent, rather than delaying action until symptoms or disease progression occur.

Evidence-based studies have shown that NUC-treated patients with low HBsAg levels and IHCs can achieve high HBsAg clearance rates with Peg-IFNα treatment (Mo et al., 2024). In clinical practice, we observed that patients with HBsAg levels below 50 IU/ml respond well to short-term Peg-IFNα treatment, a phenomenon not widely reported. This study evaluated the clinical outcomes of short-term Peg-IFNα treatment in NUC-treated patients and IHCs with ultra-low HBsAg levels, comparing them with a control group that did not receive Peg-IFNα, and aimed to identify potential influencing factors.

## 2 Materials and methods

### 2.1 Study population

This retrospective, real-world clinical study was conducted at the outpatient clinic of the ‘Infectious Diseases Department of Southwest Hospital from January 2019 to December 2022. Participants were 20–60 years old. According to China’s Guidelines for the Prevention and Treatment of Chronic Hepatitis B (2019 version*)*, eligible nucleos(t)ide analogue (NUC)-treated patients and inactive HBsAg carriers (IHCs) met the following criteria: positive HBsAg for more than 6 months, HBsAg levels <50 IU/ml, and HBeAg negative, with or without HBeAb positivity. IHCs also needed to be treatment-naïve, have normal ALT levels, and HBV DNA <2,000 IU/ml. Prior to NUC treatment, the laboratory characteristics of CHB patients included fluctuating or persistently elevated ALT levels as well as high serum HBV DNA titers (typically >2,000 IU/ml). NUC-treated patients had to receive NUC for at least 1 year, be HBeAg negative before NUC treatment or have experienced HBeAg seroconversion during treatment, with HBV DNA <2,000 IU/ml. Exclusion criteria included: (1) decompensated cirrhosis or other liver diseases (e.g., hepatocellular carcinoma, autoimmune liver diseases, alcoholic liver diseases, or drug abuse); (2) severe systemic diseases (e.g., uncontrolled hypertension, chronic kidney disease, diabetes, thyroid dysfunction, rheumatic diseases, or mental disorders); (3) co-infection with other hepatitis viruses (A, C, D, or E) or cytomegalovirus, Epstein–Barr virus, or HIV; (4) pregnancy or lactation. This study was approved by the Ethics Committee of Southwest Hospital (ethical approval number: (B) KY2024089), and all participants provided written informed consent before enrollment.

### 2.2 Research methods

Based on patient preferences, the NUC-treated cohort was split into the NUC add-on Peg-IFNα group and the NUC group. The IHC cohort was divided into the Peg-IFNα group and the untreated group. The NUC add-on Peg-IFNα group received a weekly 180 μg subcutaneous injection of Peg-IFNα (pegylated interferon alpha-2b injection, trade name: Pegbina; Xiamen Amoytop Biotech) in addition to their ongoing NUC regimen. The NUC group continued the original NUC regimen. In the Peg-IFNα group, patients with HBV DNA <20 IU/ml received subcutaneous Peg-IFNα. Those with HBV DNA between 20 and 2,000 IU/ml received a combination of subcutaneous Peg-IFNα and oral tenofovir disoproxil fumarate (TDF, 300 mg/day). The untreated group served as the control. The total Peg-IFNα treatment duration was at least 24 weeks. If HBsAg clearance occurred, consolidation treatment was given as per medical guidelines. If there was no significant decrease in HBsAg from baseline, treatment was stopped. The primary efficacy endpoint was the HBsAg clearance rate at 24 weeks. Secondary endpoints included the HBsAg clearance rate when HBsAg <10 IU/ml and the identification of predictive factors for HBsAg clearance at 24 weeks. Adverse reactions to Peg-IFNα were monitored to assess safety.

### 2.3 Research evaluation

Prior to Peg-IFNα treatment, patients were tested for HBV markers, HBV DNA, liver function, renal function, blood glucose, complete blood count, thyroid function, liver disease-related autoantibodies, and underwent upper abdominal ultrasound and FibroScan. During treatment, complete blood count, liver function, renal function, and blood glucose levels were monitored every 4 weeks. HBV markers, quantitative detection of COBAS HBV DNA, thyroid function, and liver disease-related autoantibodies were assessed every 12 weeks. HBV markers were measured using the ARCHITECT i2000SR automatic immunoassay analyzer (chemiluminescent microparticle immunoassay, Abbott Ireland Diagnostics Division). The measurement range of HBsAg concentration was 0.05 IU/ml to 250,000 IU/ml (after dilution). HBsAg clearance was defined as <0.05 IU/ml (below the lower limit of detection), and HBsAb positivity as >10 IU/L. Serum HBV DNA levels were quantified using the COBAS TaqMan automatic PCR analysis system (Roche, Switzerland), with a detection limit of 20 IU/ml. Serum ALT levels were measured using the 7600–020 automatic biochemical analyzer (Hitachi, Japan), with a normal range of 0–42 IU/ml. Safety assessments included monitoring for fever, myalgia, fatigue, alopecia, mental disturbances, reduced appetite, neutropenia, elevated ALT, thrombocytopenia, and thyroid dysfunction.

### 2.4 Statistical analysis

Data analysis was performed using SPSS version 26.0. Categorical data were presented as frequencies and percentages (%). Continuous data with a normal distribution were shown as mean ± standard deviation; those with a non-normal distribution were expressed as median and interquartile range [M (P25, P75)]. The chi-square test was used to compare HBsAg clearance and seroconversion rates. Cox regression analysis identified factors associated with HBsAg clearance. The receiver operating characteristic (ROC) curve was used to determine the optimal cut-off value for predicting HBsAg clearance at 24 weeks. A p-value <0.05 was considered statistically significant.

## 3 Results

### 3.1 Baseline characteristics

**Figure 1:**
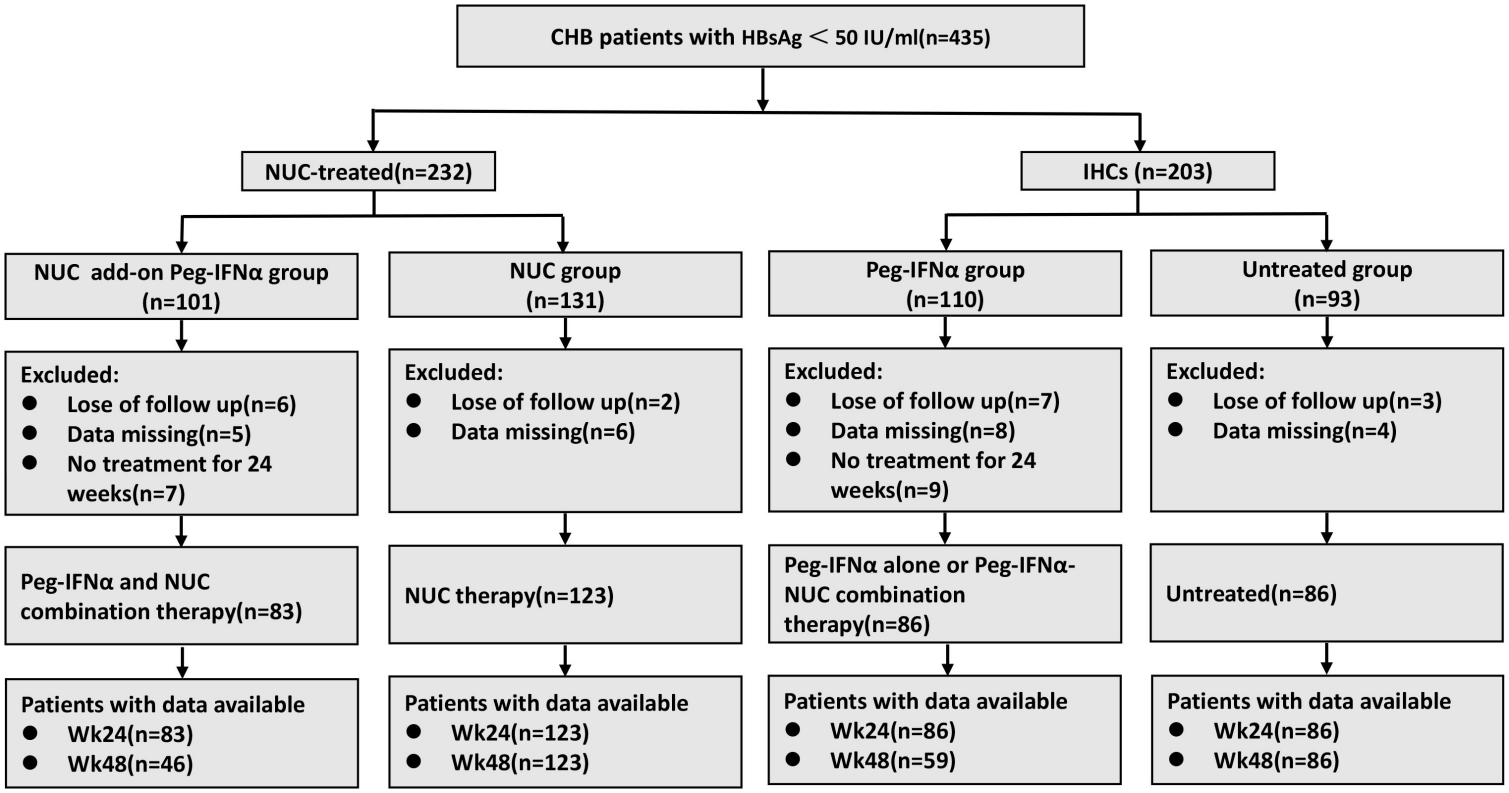
The flow diagram of patient enrollment in this study. CHB, chronic hepatitis B; NUC, nucleos(t)ide analogues; IHCs, inactive hepatitis B surface antigen carriers; Peg-IFNα, pegylated interferon alpha.

A total of 435 patients (232 NUC-treated and 203 IHCs) were recruited for this study. Of the NUC-treated patients, 101 received NUC add-on Peg-IFNα (NUC add-on Peg-IFNα group) and 131 received NUC monotherapy (NUC group). In the IHC cohort, 110 received Peg-IFNα monotherapy (Peg-IFNα group) and 93 remained untreated (untreated group). Among these patients, 18 were lost to follow-up in all groups, 23 did not undergo regular examinations resulting in data loss, and 16 discontinued treatment due to adverse events or other reasons. The final cohort for statistical analysis included 83 patients in the NUC add-on Peg-IFNα group, 123 in the NUC group, 86 in the Peg-IFNα group, and 86 in the untreated group (**Figure 1**).

Moreover, there were no statistically significant differences in baseline characteristics (including sex, age, HBsAg level, HBV DNA, NUC regimen, ALT, neutrophil count, platelets, triiodothyronine, thyroxine, and thyroid-stimulating hormone) among the four groups (**Table 1**; p > 0.05 for all).

**Table 1:** Baseline characteristics of the study population.

	NUC add-on Peg-IFNα group (n=83)	NUC Group (n=123)	Peg-IFNα group (n=86)	Untreated group (n=86)	P-Value
Male (%)	65 (78.31)	87 (70.73)	57 (66.28)	54 (62.79)	0.146
Age, mean ± SD	41.87 ± 8.15	41.50 ± 8.33	38.86 ± 8.89	40.76 ± 8.71	0.087
HBsAg at week 0					0.436
<10 IU/ml (%)	44 (53.01)	60 (48.78)	42 (48.84)	51 (59.30)	
10–50 IU/ml (%)	39 (46.99)	63 (51.22)	44 (51.16)	35 (40.70)	
HBV DNA at week 0					0.073
<20 IU/ml (%)	74 (89.16)	112 (91.06)	68 (79.07)	73 (84.88)	
20–2000 IU/ml (%)	9 (10.84)	11 (8.94)	18 (20.93)	13 (15.12)	
Different NUC					0.062
ETV (%)	52 (62.65)	92 (74.80)			
TDF (%)	31 (37.35)	31 (25.20)			
ALT, IU/L, M (P_25_, P_75_)	29.55 (20.00,39.60)	30.25 (20.48,42.08)	28.20 (17.00,36.30)	28.40 (18.85,35.25)	0.053
NEU, 10^9^/L, M (P_25_, P_75_)	3.28 (2.73, 4.02)	3.36 (2.63,4.15)	3.48 (2.58,4.04)	3.39 (2.76,4.45)	0.769
PLT, 10^9^/L, mean ± SD	187.43 ± 36.09	173.15 ± 44.61	183.66 ± 37.37	179.51 ± 38.12	0.141
T3, nmol/L, mean ± SD	1.87 ± 0.32	1.90 ± 0.68	1.96 ± 0.98	1.82 ± 0.23	0.702
T4, nmol/L, mean ± SD	104.28 ± 11.15	101.76 ± 14.51	100.21 ± 16.71	102.08 ± 14.38	0.571
TSH, uIU/ml, mean ± SD	2.14 ± 1.03	2.09 ± 0.92	2.01 ± 0.88	2.02 ± 0.90	0.861

There were no statistically significant differences in baseline characteristics (p>0.05).

### 3.2 The HBsAg clearance rate in the group treated with Peg-IFNα was significantly higher than that in the control group

**Figure 2:**
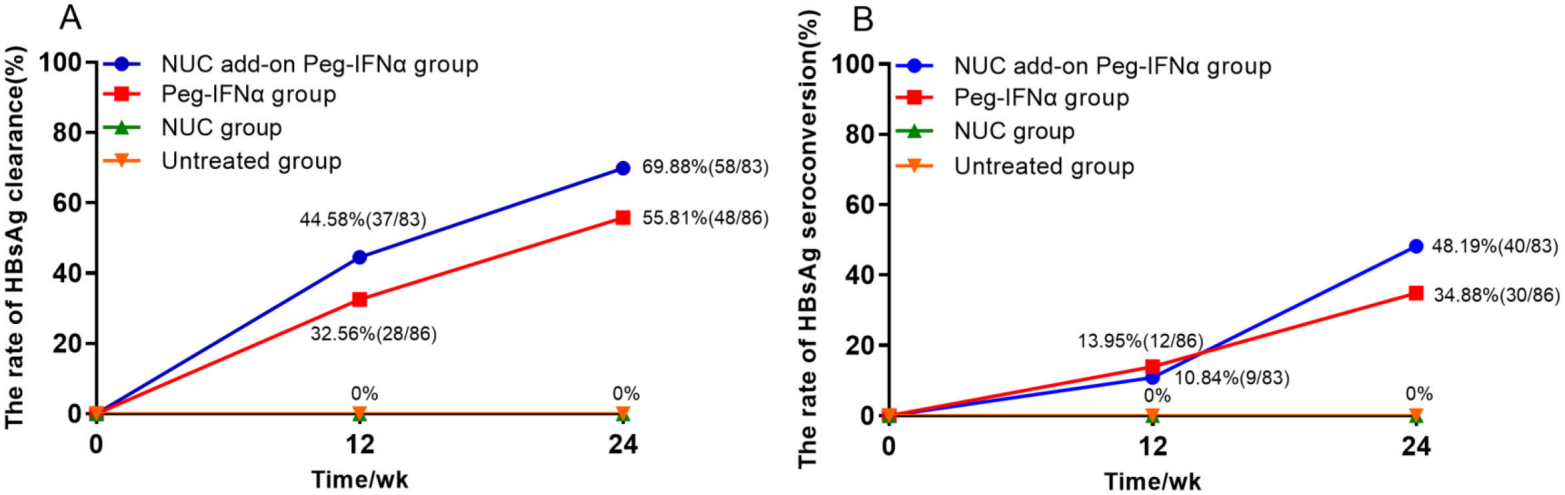
Rates of HBsAg clearance and HBsAg seroconversion. **(A)** Accumulated rates of HBsAg clearance: at 24 weeks, HBsAg clearance rates were 69.88% in the NUC add-on Peg-IFNα group and 55.81% in the Peg-IFNα group, both significantly higher than 0% in the NUC and untreated groups (p<0.001). There was no significant difference between the two Peg-IFNα groups (p=0.059). **(B)** Accumulated rates of HBsAg seroconversion: at 24 weeks, HBsAg seroconversion rates were 48.19% and 34.88% in the NUC add-on Peg-IFNα and Peg-IFNα groups, respectively, with no statistical difference (p=0.079). No seroconversion occurred in the NUC or untreated groups (p<0.001). HBsAg, hepatitis B surface antigen; Peg-IFNα, Pegylated interferon alpha; NUC, nucleos(t)ide analogues.

At week 24, the HBsAg clearance rates in the NUC add-on Peg-IFNα group and the Peg-IFNα group were 69.88% (n=58/83) and 55.81% (n=48/86), respectively. Both rates were significantly higher than the 0% observed in either the NUC group or the untreated group (**Figure 2A**; p < 0.001). Similarly, the HBsAg seroconversion rates in the NUC add-on Peg-IFNα group and the Peg-IFNα group were 48.19% (n=40/83) and 34.88% (n=30/86), respectively, both far exceeding the 0% observed in the groups without Peg-IFNα therapy (**Figure 2B**; p < 0.001). Moreover, there were no significant differences between the two Peg-IFNα treatment groups in terms of HBsAg clearance rate (**Figure 2A**; p = 0.059) or seroconversion rate (**Figure 2B**; p = 0.079).

Among the 169 Peg-IFNα-treated patients, 64 discontinued treatment at 24 weeks, with 39 (60.94%, n=39/64) achieving HBsAg clearance. Meanwhile, 105 extended treatment to 48 weeks, and 72 (68.57%, n=72/105) achieved HBsAg clearance. There was no significant difference in HBsAg clearance between the group that stopped treatment at 24 weeks and the group that extended treatment to 48 weeks (p = 0.311).

After 24 weeks of Peg-IFNα treatment, 63 patients (25 NUC-treated and 38 IHCs) remained HBsAg positive, yet all had HBV DNA below the detection threshold. Among them, 6 patients (3 NUC-treated and 3 IHCs) showed an increase in HBsAg levels at 24 weeks compared with baseline, while the remaining 57 patients showed a decrease in levels.

### 3.3 Patients with baseline HBsAg level <no><10</no> IU/ml achieved a higher HBsAg clearance rate

**Figure 3:**
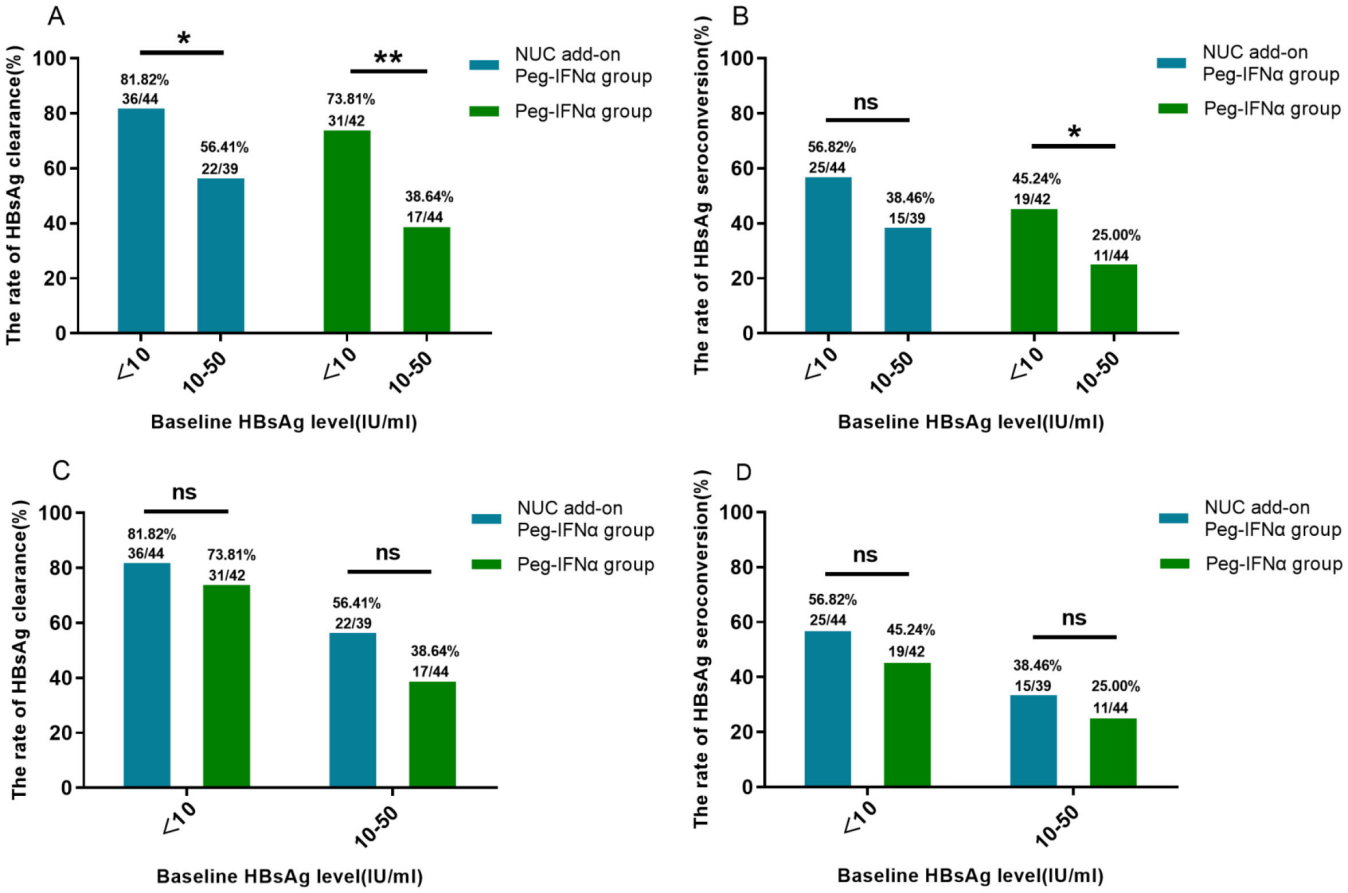
HBsAg clearance and seroconversion based on baseline HBsAg stratification. **(A)** Higher HBsAg clearance rates were observed in both the NUC add-on Peg-IFNα and Peg-IFNα groups with baseline HBsAg <10 IU/ml compared with those with baseline HBsAg 10–50 IU/ml (both p<0.05). **(B)** In the Peg-IFNα group, patients with baseline HBsAg <10 IU/ml had higher seroconversion rates (p<0.05), while no significant difference was seen in the NUC add-on Peg-IFNα group (p=0.095). **(C, D)** No significant differences in clearance or seroconversion rates were found between the NUC add-on Peg-IFNα and Peg-IFNα groups across baseline HBsAg levels (all p>0.05). HBsAg, hepatitis B surface antigen; Peg-IFNα, Pegylated interferon alpha; NUC, nucleos(t)ide analogues.

To evaluate the impact of baseline HBsAg levels on HBsAg clearance, we stratified Peg-IFNα-treated patients by baseline HBsAg levels into two groups: <10 IU/ml and 10–50 IU/ml. At 24 weeks of treatment, the HBsAg clearance rate in the <10 IU/ml group was 77.91% (n=67/86), significantly higher than 46.99% (n=39/83) observed in the 10–50 IU/ml group (p < 0.001). Similarly, the HBsAg seroconversion rate in the <10 IU/ml group was 51.16% (n=44/86), significantly greater than 31.33% (n=26/83) in the 10–50 IU/ml group (p < 0.01). When analyzing NUC-treated patients and IHCs separately, the <10 IU/ml group demonstrated a higher HBsAg clearance rate in both subgroups (**Figure 3A**). In the NUC-treated group, the clearance rate was 81.82% (n=36/44) vs. 56.41% (n=22/39) (p<0.05), while in the IHC group, it was 73.81% (n=31/42) vs. 38.64% (n=17/44) (p<0.01).

Regarding the HBsAg seroconversion rate (**Figure 3B**), among NUC-treated patients, no significant difference was observed between the <10 IU/ml group (56.82%, n=25/44) and the 10–50 IU/ml group (38.46%, n=15/39) (p = 0.095). In contrast, in the IHC group, the seroconversion rate in the <10 IU/ml group (45.24%, n=19/42) was significantly higher than that in the 10–50 IU/ml group (25.00%, n=11/44) (p<0.05). Within the same baseline HBsAg strata, no significant differences in HBsAg clearance or seroconversion rate were observed between the NUC add-on Peg-IFNα group and the Peg-IFNα group (**Figures 3C, D**; p > 0.05).

### 3.4 No significant difference in HBsAg clearance rate between Peg-IFNα monotherapy and the combination therapy of Peg-IFNα with NUC in IHCs

Among IHCs, 76 patients received Peg-IFNα monotherapy and 10 patients received Peg-IFNα and NUC combination therapy. At week 24, the HBsAg clearance rate was 55.26% (n=42/76) in the monotherapy group and 60.00% (n=6/10) in the combination group, with no significant difference (p = 1.000).

### 3.5 The decline rate of HBsAg level from baseline to week 12 as a predictor for HBsAg clearance at week 24

**Figure 4:**
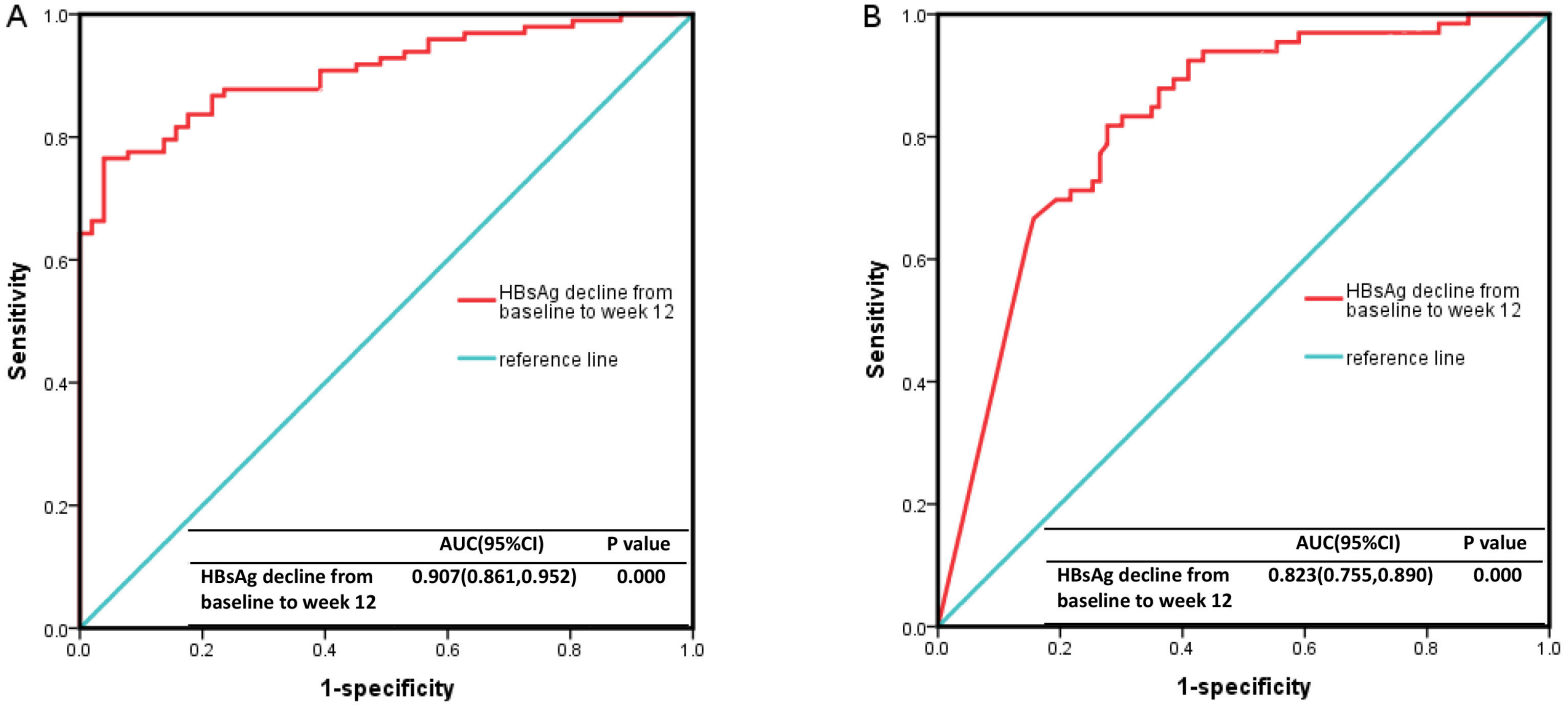
The ROC curve results. **(A)** The decline rate of HBsAg level from baseline to 12 weeks had a high predictive value for HBsAg clearance (AUC ≥0.9, cutoff value=95.8%, sensitivity 0.765, specificity 0.961). **(B)** It had a moderate predictive value for HBsAg seroconversion (0.7 ≤AUC <0.9, cutoff value=95.8%, sensitivity 0.818, specificity 0.723). ROC, Receiver Operating Characteristic curve; HBsAg, hepatitis B surface antigen.

To identify factors associated with HBsAg clearance after 24 weeks of Peg-IFNα treatment, we analyzed several variables, including sex, age, baseline HBsAg/HBV DNA levels, NUC combination therapy, HBsAg decline at week 12, and others. Univariable Cox regression analysis demonstrated that baseline HBsAg level, HBsAg decline rate at week 12, HBsAg decline at week 12, and baseline HBsAg <10 IU/ml were significant predictors of HBsAg clearance at 24 weeks (p < 0.05). Multivariable Cox regression analysis further confirmed that the HBsAg decline rate at week 12 was an independent predictor (p < 0.05) (**Table 2**). Receiver operating characteristic (ROC) curve analysis demonstrated that the area under the curve (AUC) was ≥0.9, with an optimal cut-off of 95.8%, a sensitivity of 0.765, and a specificity of 0.961 (**Figure 4A**). Therefore, a ≥95.8% decline in HBsAg level at week 12 could serve as a reliable predictor for achieving HBsAg clearance at week 24. However, its predictive value for HBsAg seroconversion was only moderate (**Figure 4B**).

**Table 2:** Cox regression analysis of HBsAg clearance risk at week 24 in all Peg-IFNα-treated patients.

Predictors	Univariable analysis		Multivariable analysis	
	HR (95%CI)	P	HR (95%CI)	P
Gender (male)	1.009 (0.652∼1.561)	0.969		
Age, years	0.982 (0.959∼1.005)	0.118		
Baseline HBV DNA, <20 IU/ml	0.682 (0.403∼1.154)	0.154		
Combination NUC or not	1.351 (0.921∼1.982)	0.123		
Baseline HBsAg level, IU/ml	0.974 (0.959∼0.989)	0.001	0.987 (0.960∼1.014)	0.340
Baseline HBsAg level <10 IU/ml	2.011 (1.354∼2.987)	0.001	1.502 (0.742∼3.042)	0.259
HBsAg level at week 12, IU/ml	0.804 (0.726∼0.890)	<0.001	0.925 (0.825∼1.037)	0.181
HBsAg decline from baseline to week 12,%	1.023 (1.013∼1.033)	<0.001	1.019 (1.007∼1.031)	0.002
HBsAg decline at week 12, IU/ml	1.006 (0.993∼1.018)	0.370		
ALT elevation from baseline to week 12, %	1.000 (1.000∼1.001)	0.268		
NEU decline from baseline to week 12, %	1.003 (0.996∼1.010)	0.441		
PLT decline from baseline to week 12, %	1.007 (0.995∼1.020)	0.245		
T3 decline from baseline to week 12, %	1.012 (0.996∼1.027)	0.139		
T4 decline from baseline to week 12, %	1.008 (0.995∼1.021)	0.217		
TSH decline from baseline to week 12, %	1.001 (0.996∼1.006)	0.699		

Univariate Cox regression analysis demonstrated that baseline HBsAg level, HBsAg decline rate at week 12, HBsAg decline at week 12, and baseline HBsAg <10 IU/ml were significant predictors of HBsAg clearance at 24 weeks (p<0.05). Multivariable Cox regression analysis further confirmed that the HBsAg decline rate at week 12 was an independent predictor (p<0.05).

### 3.6 Follow-up

In the NUC add-on Peg-IFNα group receiving Peg-IFNα treatment, 37 patients stopped at 24 weeks and 46 extended to 48 weeks. Among 22 patients followed to 48 weeks after stopping at 24 weeks, 2 additional HBsAg clearances, 3 HBsAg seroconversions, and 3 cases of HBsAg re-positivity occurred. Of these, 17 were followed to 96 weeks, with 2 more seroconversions and 1 case of HBsAg re-positivity, but no further HBsAg clearances. Among those extending to 48 weeks, 1 additional HBsAg clearance and 4 more HBsAg seroconversions were observed. Of 32 patients followed to 96 weeks, 1 more seroconversion and 2 cases of HBsAg re-positivity occurred, with no further HBsAg clearances.

In the Peg-IFNα group undergoing Peg-IFNα therapy, 27 patients stopped at 24 weeks and 59 extended to 48 weeks. Among 20 patients followed to 48 weeks after stopping at 24 weeks, 3 additional HBsAg clearances, 4 HBsAg seroconversions, and 1 case of HBsAg re-positivity occurred. Of these, 14 were followed to 96 weeks, with 1 case of HBsAg re-positivity but no further HBsAg clearances or seroconversions. Among those extending to 48 weeks, 4 additional HBsAg clearances and 6 more HBsAg seroconversions were observed. Of 39 patients followed to 96 weeks, 3 more HBsAg clearances, 3 HBsAg seroconversions, and 5 cases of HBsAg re-positivity occurred.

**Figure 5:**
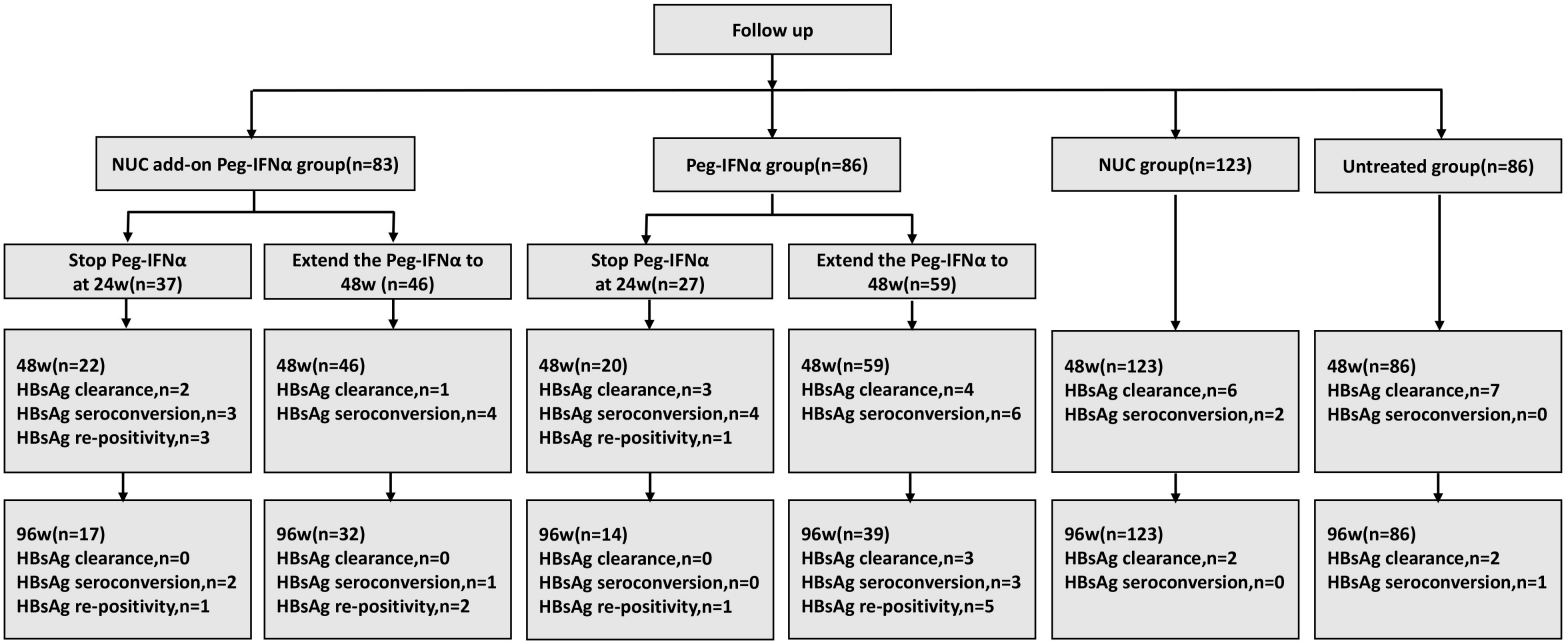
Patients’ follow-up information. NUC, nucleos(t)ide analogues; IHCs, inactive hepatitis B surface antigen carriers; Peg-IFNα, pegylated interferon alpha.

In the NUC group, among 123 patients followed to 48 weeks, 6 HBsAg clearances (including 2 with HBsAg seroconversions) were recorded. At the 96-week follow-up, 2 more HBsAg clearances occurred, with no additional HBsAg seroconversions. In the untreated group, among 86 patients, 7 HBsAg clearances occurred without seroconversions (all with baseline HBsAg levels of 0.05–1 IU/ml). At the 96-week follow-up, 2 more HBsAg clearances (including 1 with HBsAg seroconversion) occurred (**Figure 5**).

### 3.7 Adverse events

In this study, common adverse reactions to Peg-IFNα included fever, myalgia, fatigue, alopecia, anxiety, loss of appetite, neutropenia, elevated ALT levels, thrombocytopenia, and thyroid dysfunction (**Table 3**). The frequencies of adverse events were similar between the NUC add-on Peg-IFNα group and the Peg-IFNα group (p > 0.05). Notably, only one patient experienced severe thyroid hyperthyroidism requiring medical intervention, and patients generally tolerated Peg-IFNα well. Adverse reactions gradually subsided after discontinuation of treatment. In addition, due to the absence of significant adverse events in the overall NUC group, no obvious differences in adverse events were identified between patients receiving different types of NUC. No patient developed cirrhosis or hepatocellular carcinoma (HCC) during the study period.

**Table 3:** Adverse events associated with Peg-IFNα and NUC.

Adverse events	Total (n=169)	NUC add-on Peg-IFNα group (%,n) (n=83)	Peg-IFNα group (%,n) (n=86)
Pyrexia	69.23 (117)	68.67 (57)	69.77 (60)
Myalgia	40.23 (68)	38.55 (32)	41.86 (36)
Fatigue	31.95 (54)	30.12 (25)	33.72 (29)
Alopecia	23.67 (40)	22.89 (19)	24.42 (21)
Anxiety	17.16 (29)	16.87 (14)	17.44 (15)
Decreased appetite	24.85 (42)	24.10 (20)	25.58 (22)
Neutropenia	72.78 (123)	67.47 (56)	77.91 (67)
ALT elevation	75.15 (127)	77.11 (64)	73.26 (63)
Thrombocytopenia	67.46 (114)	69.88 (58)	65.12 (56)
Thyroid dysfunction	8.88 (15)	8.43 (7)	9.30 (8)

Number and proportion of patients who experienced adverse events while receiving Peg-IFNα or combination therapy with NUC.

## 4 Discussion

The ultimate goal of CHB treatment is to delay and reduce the occurrence of adverse outcomes such as liver cirrhosis, HCC, and liver failure. This is currently a priority clinical issue to be addressed. Therefore, achieving functional cure plays a crucial role in realizing these objectives (Vittal et al., 2022; Yip et al., 2021; Song et al., 2021b).

HBsAg clearance is key to functional cure and significantly reduces the risk of long-term complications in HBV-infected individuals (Anderson et al., 2021; Pan et al., 2021). For eligible patients, achieving HBsAg clearance should be prioritized (Chinese Medical Association and Chinese Society of Hepatology, Chinese Society of Infectious Diseases, Chinese Medical Association, 2022). Peg-IFNα, which is more effective than NUC in promoting HBsAg clearance, is preferred for patients without contraindications. However, compared with oral NUC, its use is limited by high costs, subcutaneous administration, and potential side effects, leading to lower patient acceptance (Wong et al., 2022). Therefore, developing effective short-term regimens for patients with low baseline HBsAg levels is of considerable importance, as it can reduce the economic burden and minimize the duration of exposure to side effects.

This study aimed to compare the efficacy of Peg-IFNα in achieving HBsAg clearance between NUC-experienced patients and IHCs with ultra-low HBsAg levels in a real-world setting, using Peg-IFNα-free patients as controls. Previous studies targeting functional cure typically employed a 48-week Peg-IFNα regimen, with limited evidence on the efficacy of shorter courses. In NUC-experienced patients, 24 weeks of Peg-IFNα combined with NUC resulted in HBsAg clearance rates of 28.71% and 42.86% for baseline HBsAg levels ≤1,000 IU/ml and ≤100 IU/ml, respectively (Zhang et al., 2023). The Everest Project in China reported a 36.7% HBsAg clearance rate after 24 weeks of treatment for baseline HBsAg <500 IU/ml (Xie et al., 2022). Similarly, two studies on IHCs demonstrated HBsAg clearance rates of 30% and 39.50% for baseline HBsAg <1,000 IU/ml, and 49.12% for HBsAg ≤100 IU/ml after 24 weeks of treatment (Zhang et al., 2023; Lim et al., 2024). In our study, 24 weeks of Peg-IFNα combined with NUC achieved HBsAg clearance and seroconversion rates of 69.88% and 48.19%, respectively, significantly higher than the zero clearance and seroconversion rates observed in the NUC-only group (p < 0.001). In the IHC group, the HBsAg clearance and seroconversion rates were 55.81% and 34.88%, respectively, also significantly higher than the untreated group (p < 0.001). The higher HBsAg clearance rates observed in both groups may be attributed to the ultra-low baseline HBsAg levels and HBeAg-negative status of the enrolled patients. The likelihood of spontaneous HBsAg clearance increases over time in populations with ultra-low HBsAg levels (Barone et al., 2023). At the 48- and 96-week follow-ups, HBsAg clearance rates increased in both the NUC group (4.87% [n=6/123] to 6.50% [n=8/123]) and the untreated group (8.13% [n=7/86] to 10.46% [n=9/86]). Notably, all patients achieving HBsAg clearance at 48 weeks in these groups had baseline HBsAg <1 IU/ml, yet none achieved clearance with NUC alone or without treatment at 24 weeks (**Figure 2A**). Age is an independent factor for histological progression in CHB patients (Göbel et al., 2011; Gui et al., 2010). Patients achieving HBsAg clearance before age 50 exhibit a significantly lower risk of developing HCC than those aged ≥50 years (Yip et al., 2017). These findings underscore the necessity for proactive HBsAg clearance strategies in CHB patients, even with low HBsAg levels, to maximize long-term benefits through early intervention.

In CHB patients achieving functional cure, NUC-treated patients and IHCs with low HBsAg levels exhibit comparable serological profiles. Previous studies reported 24-week HBsAg clearance rates of 36.8% and 32.6% in NUC-treated patients and IHCs with baseline HBsAg <1,000 IU/ml, respectively (Zhang et al., 2023), while 48-week rates were 52.9% and 65.5%. Our study further demonstrated similar 24-week HBsAg clearance rates in NUC-treated patients and IHCs with HBsAg <50 IU/ml (69.88% vs. 55.81%, respectively). These findings suggest equivalent treatment efficacy for HBsAg clearance in these subgroups.

Our findings underscore that baseline HBsAg levels are a key determinant of HBsAg clearance. Using a Markov model, pharmacoeconomic evaluations identified CHB patients with HBsAg <10 IU/ml as a cost-effective population for Peg-IFNα treatment (Li R, et al., 2021). Our stratified analysis further confirmed this, showing that patients with baseline HBsAg <10 IU/ml achieved a significantly higher 24-week HBsAg clearance rate (77.91%) (**Figure 3**)—consistent with the notion that lower baseline HBsAg levels strongly correlate with higher clearance rates and highlighting the critical predictive value of baseline HBsAg.

Extending Peg-IFNα therapy beyond the standard 48-week regimen was previously considered effective for improving functional cure rates (Li M, et al., 2021; Bao et al., 2020). However, our data demonstrate no additional benefit in HBsAg clearance rates when prolonging treatment from 24 to 48 weeks, with minimal relapse post-treatment. Similar observations exist across studies: in patients with HBsAg <200 IU/ml, 24-week Peg-IFNα treatment achieved a 52.1% HBsAg clearance rate, and extending treatment to 48 weeks had no effect (Li et al., 2024). For HBsAg <100 IU/ml, >70% achieved HBsAg clearance within 24 weeks, and relapses were rare (Wen et al., 2024). Higher rates (83.3%–93.8%) were reported in patients with HBsAg <20 IU/ml (Zeng et al., 2020; Zhao et al., 2020). These findings consistently indicate that baseline HBsAg levels inversely correlate with HBsAg clearance rates under 24-week Peg-IFNα therapy. Therefore, an individualized treatment course based on HBsAg levels, rather than fixed regimens, may optimize functional cure outcomes.

Multiple studies confirm that baseline HBsAg levels reliably predict functional cure in CHB patients treated with Peg-IFNα. A meta-analysis indicates an inverse relationship between baseline HBsAg levels (<3.5 log_10_ IU/ml) and HBsAg clearance rates (Zhang et al., 2024). In our study, focusing on patients with extremely low baseline HBsAg levels, the 12-week HBsAg decline rate strongly predicted 24-week HBsAg clearance. This result is in accordance with previous reports (Moucari et al., 2009; Marcellin et al., 2013). While prior studies identified a 12-week ALT increase (≥2×upper limit of normal) as an independent predictor of HBsAg clearance (Wu et al., 2021), our study found no such association, possibly due to differences in baseline characteristics or immune status among enrolled populations.

Adverse reactions associated with Peg-IFNα treatment should not be underestimated. Besides flu-like symptoms, neutropenia, thrombocytopenia, and abnormal ALT levels were common side effects. In our cohort, although the incidence of thyroid dysfunction was less than 10%, one patient developed drug-induced hyperthyroidism, necessitating intervention. After 24 weeks of treatment, the patient’s thyroid function normalized.

Notably, our data also revealed spontaneous HBsAg clearance in the untreated group: 8.13% (n=7/86) at 48 weeks and 10.46% (n=9/86) at 96 weeks of follow-up (**Figure 5**), with all 9 spontaneously clearing patients having baseline HBsAg levels between 0.05 and 1 IU/ml. This favorable spontaneous clearance trend in the extremely low HBsAg subgroup emphasizes the need for careful clinical judgment when considering active intervention, the specific role of which in enhancing clearance beyond the baseline effect remains to be clarified in future studies. In such deliberations, the potential adverse events of Peg-IFNα must be weighed alongside potential benefits, with patient well-being and preferences placed at the forefront—especially given the possibility of spontaneous clearance in this subgroup. Future research should therefore focus on two key areas: first, defining optimal HBsAg cut-offs to guide stratified treatment strategies for NUC-treated patients and IHCs; second, clarifying the specific role of Peg-IFNα in these subgroups to maximize benefits while minimizing unnecessary burdens and risks.

This study has several limitations. First, it is a single-center, retrospective study with a small sample size. The non-randomized design, which was based on patient preferences, gave rise to selection bias. Second, treatment discontinuation and loss to follow-up among patients who extended Peg-IFNα therapy could affect the accuracy of efficacy evaluation and factor analysis. Finally, long-term follow-up is needed to assess sustained HBsAg clearance and liver disease progression. Future large-scale, multi-center studies are required to validate these findings.

In conclusion, our study shows that 24-week Peg-IFNα treatment can significantly augment HBsAg clearance rates in both NUC-treated patients and IHCs with baseline HBsAg levels <50 IU/ml. Notably, the efficacy is comparable between the two groups. This short-term regimen enables patients to achieve HBsAg clearance at an earlier stage.

## Data Availability

The original contributions presented in the study are included in the article/supplementary material. Further inquiries can be directed to the corresponding authors.
